# When Competitors Join Forces: Consortia of Entomopathogenic Microorganisms Increase Killing Speed and Mortality in Leaf- and Root-Feeding Insect Hosts

**DOI:** 10.1007/s00248-023-02191-0

**Published:** 2023-02-27

**Authors:** Anna Spescha, Maria Zwyssig, Mathias Hess Hermida, Aurélie Moix, Pamela Bruno, Jürg Enkerli, Raquel Campos-Herrera, Giselher Grabenweger, Monika Maurhofer

**Affiliations:** 1 Institute of Integrative Biology, ETH Zurich, Zurich, Switzerland; 2 Research Group Extension Arable Crops, Agroscope, Zurich, Switzerland; 3 Division of Agricultural Entomology, Department of Crop Sciences, Georg-August-Universität Göttingen, Göttingen, Germany; 4 Research Group Molecular Ecology, Agroscope, Zurich, Switzerland; 5 Instituto de Ciencias de la Vid y del Vino (ICVV), CSIC, Universidad de La Rioja, Gobierno de La Rioja, Logroño, Spain

**Keywords:** Biocontrol consortia, Insecticidal pseudomonads, Entomopathogenic nematodes, Entomopathogenic fungi, Co-infections, Interaction of biocontrol organisms

## Abstract

Combining different biocontrol agents (BCA) is an approach to increase efficacy and reliability of biological control. If several BCA are applied together, they have to be compatible and ideally work together. We studied the interaction of a previously selected BCA consortium of entomopathogenic pseudomonads (*Pseudomonas chlororaphis*), nematodes (*Steinernema feltiae* associated with *Xenorhabdus bovienii*), and fungi (*Metarhizium brunneum*). We monitored the infection course in a leaf- (*Pieris brassicae*) and a root-feeding (*Diabrotica balteata*) pest insect after simultaneous application of the three BCA as well as their interactions inside the larvae in a laboratory setting. The triple combination caused the highest mortality and increased killing speed compared to single applications against both pests. Improved efficacy against *P. brassicae* was mainly caused by the pseudomonad-nematode combination, whereas the nematode-fungus combination accelerated killing of *D. balteata*. Co-monitoring of the three BCA and the nematode-associated *Xenorhabdus* symbionts revealed that the four organisms are able to co-infect the same larva. However, with advancing decay of the cadaver there is increasing competition and cadaver colonization is clearly dominated by the pseudomonads, which are known for their high competitivity in the plant rhizosphere. Altogether, the combination of the three BCA increased killing efficacy against a Coleopteran and a Lepidopteran pest which indicates that this consortium could be applied successfully against a variety of insect pests.

**Graphical Abstract:**

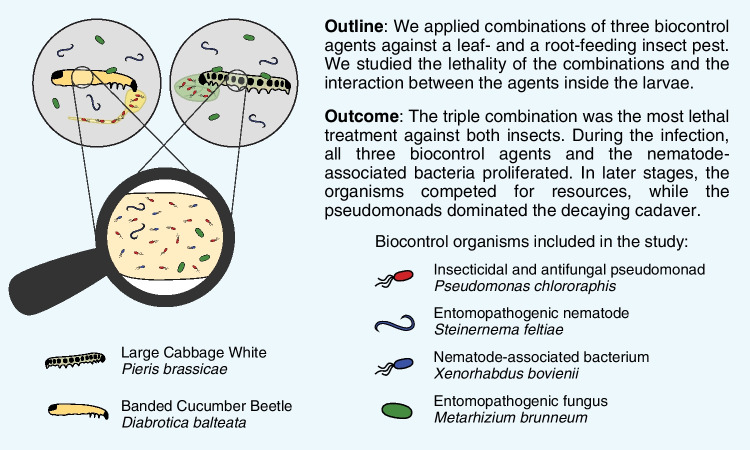

**Supplementary Information:**

The online version contains supplementary material available at 10.1007/s00248-023-02191-0.

## Introduction

Global food production relies heavily on synthetic pesticides to protect crops from pathogens and pests [1]. The pressure to limit pesticide use and the demand for alternative control solutions are increasing [2]. One alternative is biological control, which is the “use of living organisms to suppress the population density or impact of a specific pest organism, making it less abundant or less damaging than it would otherwise be” [3]. For some diseases, biocontrol solutions are widely utilized, yet for many pathogens and pests, efficient biocontrol products are not available [4]. Despite numerous success stories [2, 5, 6], the unstable performance of biocontrol agents (BCA) is a great challenge for reliable biocontrol solutions [7]. One approach to increase biocontrol efficacy is to combine different biocontrol agents with different modes of action and ecological niches [8]. Several studies found improved biocontrol success when applying BCA consortia [9–11]. However, other studies have reported antagonistic interactions when applying combinations of microbial BCA [12]. It is crucial to assess compatibility of selected organisms in order to develop an efficient BCA consortium.

Interactions between BCA can increase the efficacy of the consortium, but BCA can also negatively impact the other consortium members. Competition for nutrients and space could lead to inhibition; toxins and antimicrobial compounds produced by microbial BCA may affect the consortium partners or the defense reaction of the host. In this study, we explored the interactions within a consortium of three biocontrol agents, namely, entomopathogenic pseudomonads (EPP), nematodes (EPN), and fungi (EPF). EPP from the species *Pseudomonas chlororaphis* are root-colonizing bacteria with plant-growth promoting, antifungal, and insecticidal properties [13–15]. Their oral insecticidal activity largely relies on multiple toxins, enzymes, and antimicrobial exoproducts [16, 17]. The studied EPN species *Steinernema feltiae* is associated with entomopathogenic *Xenorhabdus bovienii* (nematode-associated bacteria, NB) [18]. The bacteria are released in the insect hemocoel by the nematodes where they multiply and kill the insect with toxins and antimicrobials, though the nematodes themselves also contribute with own toxins [19, 20]. When resources are used up after several cycles of nematode reproduction, the nematodes take up bacteria and form a free-living infective juvenile (IJ) stage to hunt for a new host [18]. Finally, the EPF member of the consortium is *Metarhizium brunneum*, a common organism in agricultural soils especially in temperate regions [21]. *M. brunneum* infects and kills insects by attaching to and breaching through the cuticle, colonizing the insect hemolymph, and producing different proteases, toxins, and exoproducts during the whole process [22, 23]. According to earlier studies, biocontrol combinations of EPF and EPN can have additive, synergistic and, rarely, also antagonistic effects [24]. For EPP and EPN, recent publications report frequent interactions during EPN infections, and it was proposed that EPP belong to the EPN pathobiome [25, 26].

In a previous study, we have investigated the biocontrol effect of a *P. chlororaphis*-*S. feltiae*-*M. brunneum* consortium against the cabbage maggot *Delia radicum* in pot and field experiments. Our results indicated that these BCA do not impede each other’s survival in the soil or in the rhizosphere [27]. However, we know little about how they interact with and affect each other while infecting the same host. Therefore, our aim in this study was to explore the interactions between these EPP, EPF, and EPN in mixed infections and to examine the effect of different combinations on the host insect and the BCA themselves. We used single BCA and different combinations thereof to infect larvae of the large cabbage white (LCW) *Pieris brassicae* (Lepidoptera: Pieridae), an important pest feeding on above-ground plant parts of Brassicacean crops, and the root-feeding banded cucumber beetle (BCB) *Diabrotica balteata* LeConte (Coleoptera: Chrysomelidae), a sister species of the highly devastating western corn rootworm *D. virgifera virgifera*. While monitoring larval mortality over time, our main focus was to observe performance and proliferation of the three BCA and the EPN-associated *Xenorhabdus* bacteria, i.e., four entomopathogens, inside their insect hosts. We hypothesize that the combined BCA application is more efficient in killing insects compared to single infections but that the BCA compete in the cadaver for resources and might hinder each other’s proliferation. This study allowed us to gain new insight into the interaction dynamics between a nematode, a bacterial, and a fungal biocontrol agent.

## Methods

### Rearing of Organisms

Eggs of the large cabbage white (LCW) *Pieris brassicae* were obtained from the Biocommunication Group (ETH Zurich, Switzerland). Larvae were fed on Savoy cabbage and kept at 25 °C (16 h, 12 kLux), 20 °C (8 h, dark), and 60% rH during rearing and experiments (see Supplementary Methods). Eggs of the banded cucumber beetle (BCB) *Diabrotica balteata* were received from Syngenta AG (Stein, Switzerland) and reared on maize seedlings (variety Damaun KS, sativa, Switzerland) at 28 °C. Experiments were conducted at 25 °C in the dark at 60% rH (see Supplementary Methods).

EPP *P. chlororaphis* PCLRT03-gfp and PCLRT03-mturq (Table 1) were stored in 44% glycerol at − 80 °C and grown for 3 days on King’s B agar [28] supplemented with cycloheximide 100 mg/l, chloramphenicol 13 mg/l, and gentamycin 10 mg/l at 24 °C. Bacteria were incubated overnight in Lysogeny broth (LB) [29] at 24 °C and 180 rpm for LCW experiments, but harvested directly from King’s B plates for BCB experiments [27]. Bacteria were washed with ddH_2_O and the concentration adjusted measuring optical density at 600 nm (OD_600_) (Genesys150, Thermo Fisher Scientific, MA, USA) with an OD_600_ of 0.1 corresponding to 10^8^ cfu/ml. Approx. 300 ml suspension were prepared in a glass beaker with 2.5 × 10^8^ cfu/ml and 5 × 10^8^ cfu/ml for experiments with LCW and BCB, respectively.

**Table 1:** Entomopathogens used in this study

Species	Strain	Origin	Reference	Experiment
*Pseudomonas chlororaphis*	PCLRT03	Potato root, Switzerland	Vesga et al. [33]	-
*P. chlororaphis*	PCLRT03-gfp	Derivative of PCLRT03, PCLRT03::miniTn7-gfp2; Gm^R^	Spescha et al. [27]	Time-shift 1–3, LCW 1–4
*P. chlororaphis*	PCLRT03-mturq	Derivative of PCLRT03, PCLRT03::miniTn7-mturquoise2; Gm^R^	This study; provided by Jordan Vacheron (University of Lausanne, Switzerland)	LCW 5, BCB 1–4
*Steinernema feltiae*	RS5 (RS-5, wild-type)	Soil, wheat field, Switzerland	Jaffuel et al. [34]	Time-shift 1–3, LCW 1–4
*Xenorhabdus bovienii*	SM5	*Steinernema feltiae* RS5	Provided by Ricardo Machado (University of Neuchâtel, Switzerland)	-
*X. bovienii*	SM5-mcherry	Derivative of SM5, SM5::16S-mcherry; Kan^R^	Provided by Alice Regaiolo (Johannes Gutenberg-University, Mainz, Germany)	-
*S. feltiae*	RS5-mche	RS5 re-associated with SM5-mcherry	This study	LCW 5, BCB 1–4
*Metarhizium brunneum*	Bip5 (BIPESCO5 /F52, wild-type)	*Cydia pomonella*, Austria	European Food Safety Authority [35]	Time-shift 1–3, LCW 1–4
*M. brunneum*	Bip5-gfp	Derivative of BIPESCO5, Bip5::pK2-BAR-egfp; glufosinate^R^	Provided by Jürg Enkerli	LCW 5, BCB 1–4

**Fig. 1:**
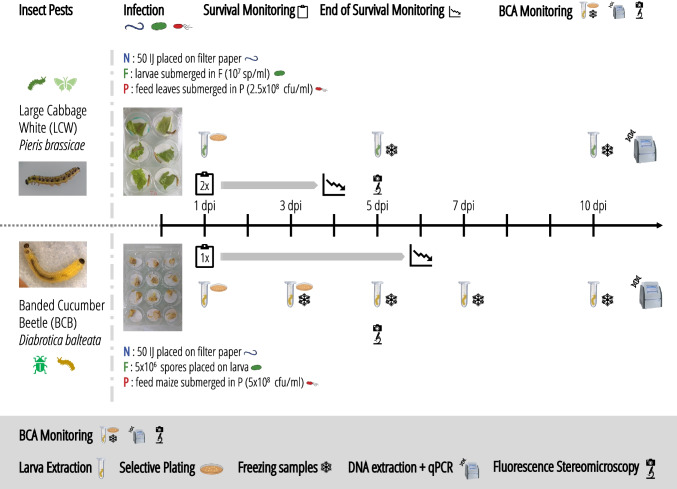
Experimental procedure for co-infection experiments with EPN, EPF, and EPP in *P. brassicae* (LCW) and *D. balteata* (BCB) larvae. LCW and BCB larvae were infected with different BCA and BCA proliferation was monitored in repetition 5 of the LCW experiment and in repetition 2 of the BCB experiment. Larvae were infected with infective juveniles (IJ) of the EPN *S. feltiae* RS5-mche (N), EPF *M. brunneum* Bip5-gfp (F), and EPP *P. chlororaphis* PCLRT03-mturq (P). Survival of LCW larvae was monitored twice a day for 4 days and once daily during 6 days for BCB larvae. For the LCW experiment, six larvae per treatment (control *n* = 3) were extracted alive at 1 day post inoculation (dpi) and dead at 5 and 10 dpi. For the BCB experiment, three alive larvae were extracted at 1 dpi, eight larvae (4 alive, 4 dead; control *n* = 4) at 3 dpi, and six (control *n* = 3) dead larvae at 5, 7, and 10 dpi. Larval extracts were plated on selective medium at 1 and 3 dpi. At 3, 5, 7, and 10 dpi, larvae were frozen for subsequent DNA extraction. Pictures of six dead larvae per treatment were taken at 5 dpi in both insect species using a fluorescence stereomicroscope. For a detailed description, see the Supplementary Methods and Table S1

Experiment indicates in which experiments and repetition a strain was used. Time-shift refers to experiments in which EPP x EPN were applied individually and in combination with a time-shift; LCW refers to experiments in which all three biocontrol agents (EPP, EPN, EPF) were applied single and in combination against the large cabbage white *P. brassicae*; BCB refers to likewise experiments conducted with the banded cucumber beetle *D. balteata*; the numbers refer to the repetition of the respective experiment. Details about monitoring BCA in different experiments are provided in Fig. 1, the Supplementary Methods, and Table S1

EPF *M. brunneum* Bip5 (wild-type) and Bip5-gfp (Table 1) were grown on selective medium (SM) agar [30] for ten days at 24 °C in the dark. Conidiospores were scraped off plates using a Drigalski glass spatula, dissolved in Tween 80 0.01%, and washed once in ddH_2_O. For LCW experiments, 20 ml of 10^7^ spores/ml were prepared in a 50-ml beaker with ddH_2_O. For BCB experiments, 10 ml of 2 × 10^8^ spores/ml were prepared in a 25-ml beaker.

EPN *S. feltiae* RS5 (wild-type, Table 1) were multiplied in *Galleria mellonella* (Lepidoptera: Pyralidae) larvae (Hebeisen fisher store, Zurich, Switzerland) at 22 °C using the White trap method [31, 32]. From *G. mellonella* cadaver infested with RS5, *X. bovienii* SM5 was isolated and tagged with mcherry and a kanamycin resistance cassette. SM5-mcherry was re-associated with *Steinernema feltiae* RS5 by injecting SM5-mcherry and kanamycin into *G. mellonella* larvae infected by RS5, and the emerging IJ population was called RS5-mche (Table 1, see Supplementary Methods). For LCW experiments, 30 ml of 1000 IJ/ml tap water were prepared, whereas 2000 IJ/ml were prepared for the BCB experiments.

### Experimental Set-Up

Larvae (3rd instar LCW and 2nd instar BCB) were starved for 6 h before use. LCW larvae were placed individually onto one ø 32-mm filter paper disk (Whatman, Huberlab, Switzerland) per well of a 6-well plate (CELLSTAR®, Greiner Bio-One, Austria). BCB larvae were placed onto two ø 20-mm filter paper disks (Whatman, Huberlab, Switzerland) per well of a 12-well plate (CELLSTAR®) (Fig. 1). Plates with BCB were sealed with a lid and 2 layers of Breathe-Easy sealing membrane (Diversified Biotech, MA, USA) to avoid escapes.

For triple infection experiments with LCW, larvae were submerged for 5 s in EPF suspension (ddH_2_O as control), 50 µl EPN suspension was pipetted on the filter paper (tap water as control), and larvae were fed with Chinese cabbage (*Brassica rapa* subsp. *pekinensis*) leaf discs previously submerged for 30 min in EPP suspension (in ddH_2_O as control). For EPP-EPN time-shift application experiments, EPN were added 6 h before (t-6 h), simultaneously (t0), or 6 h after (t + 6 h) EPP infection.

For BCB infection experiments, 25 µl EPF suspension (or ddH_2_O) and 25 µl EPN suspension (or tap water) were pipetted on the filter paper and larvae were fed with maize seedlings submerged for 60 min in EPP suspension (or in ddH_2_O).

Survival was monitored twice a day for 3 days for LCW and once a day for 6 days for BCB. For EPP-EPN-time-shift experiments, EPP colonization was determined by selective plating at 1 day post infection (dpi) and EPN proliferation was estimated by the White trap method [32] at the end of the experiment in repetitions 2 and 3. For LCW, colonization was assessed by selective plating at 1 dpi and by qPCR at 5 and 10 dpi in repetition 5. For BCB, BCA colonization was assessed by selective plating at 1 and 3 dpi and by qPCR at 3, 5, 7, and 10 dpi in repetition 2. At 5 dpi, deceased larvae were photographed under a fluorescence stereomicroscope filtering for the fluorophores of the respective strain-tag. For survival and BCA monitoring, 18 (time-shift), 24 (LCW), and 72 (BCB) larvae per treatment and repetition were prepared (see Fig. 1 and the Supplementary Methods for more details).

### Statistical Analysis

Data were analyzed with Rstudio (version 1.4.1717) using R (version 4.1.2). Pooled data on larval survival was analyzed using a cox model controlling for repetition effects, and emmeans was used for post-hoc pairwise testing (packages coxme and emmeans). Larval survival was additionally analyzed for each repetition individually using log-rank and pairwise survival difference tests (packages survival and survminer). Larval colonization by BCA was compared using ANOVA and TukeyHSD tests.

## Results

### Combinations of EPP, EPN, and EPF Killed Larvae Faster and Increased Mortality

**Fig. 2:**
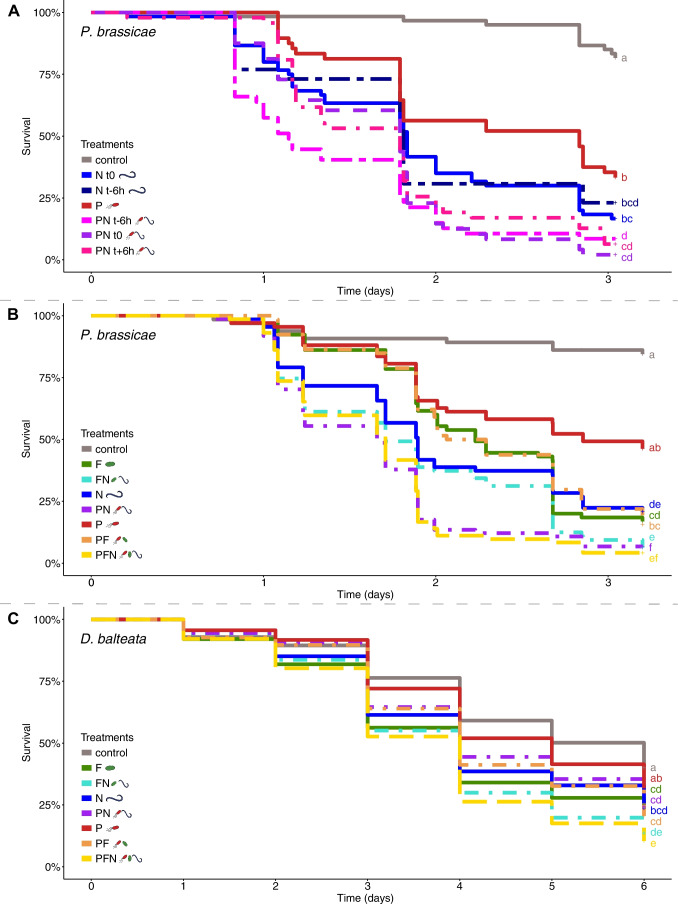
Survival of *P. brassicae* (LCW) and *D. balteata* (BCB) larvae after infection with single and combined applications of EPP, EPF and EPN. **A** EPP x EPN time-shift (ts) application experiment: t-6 h = EPN applied 6 h before EPP, t0 simultaneous application of EPP and EPN, t + 6 h EPN applied 6 h after EPP. **B** LCW experiment with single and combined simultaneous EPP, EPF, and EPN applications. **C** BCB experiment with single and combined simultaneous EPP, EPF, and EPN applications. Treatments: control = no BCA application, P = EPP *P. chlororaphis* PCLRT03-gfp or PCLRT03-mturq, N = EPN *S. feltiae* RS5 or RS5-mche, F = EPF *M. brunneum* Bip5 or Bip5-gfp, FN, PN, PF and PFN = double and triple combinations of respective BCA. Survival curves represent pooled data from three (ts 1–3) or four (LCW 1–4 and BCB 1–4) independent repetitions with 18 LCW and 60 BCB larvae per treatment and repetition. Different letters to the right of the survival curves indicate significant differences among treatments at *P* < 0.05 among pooled data. Data of individual repetitions of the three experiments (mean survival, final mortality, and statistical analysis of the survival curves) are displayed in Tables S1, S3–S4 and survival curves of individual experiments are shown in Figs. S1-S4

**Fig. 3:**
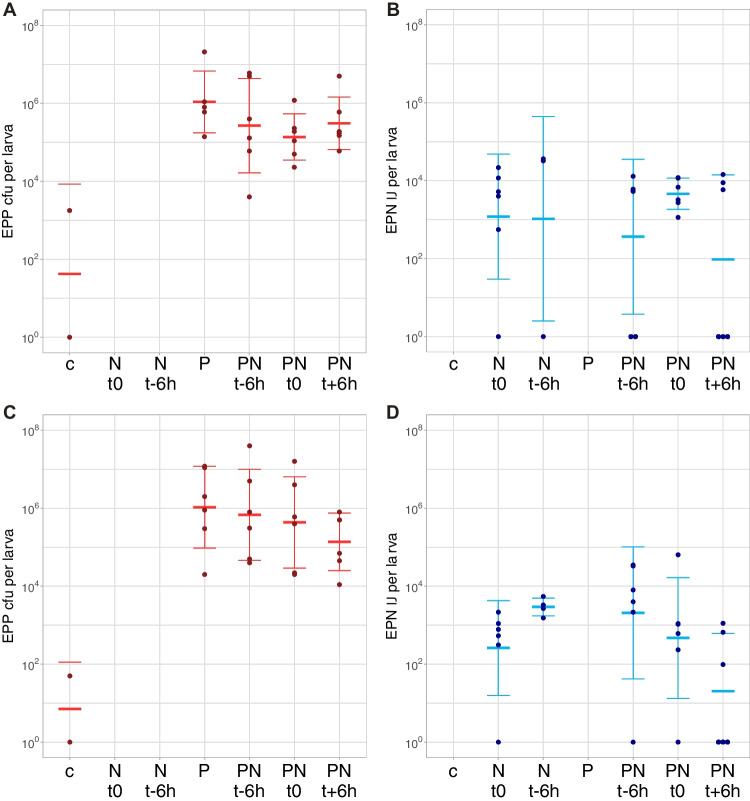
Proliferation of EPN and EPP in *P. brassicae* (LCW) larvae. Infective juvenile emergence (IJ per larva) and *Pseudomonas* colonization (cfu per larva) were assessed in the EPP x EPN time-shift application experiment for repetition 2 (**A**, **B**) and repetition 3 (**C**, **D**). Treatments: c = control with no BCA application, N t0 = EPN *S. feltiae* RS5, N t-6 h = EPN applied 6 h earlier, P = EPP *P. chlororaphis* PCLRT03-gfp, PN t-6 h = EPN applied 6 h before EPP, PN t0 = EPN and EPP applied simultaneously, PN t + 6 h = EPN applied 6 h after EPP. Left (A, C): six alive larvae were homogenized at 1 dpi and plated on selective medium and values are displayed as colony forming units (cfu) per larva. Right (B, D): six dead larvae were transferred on White traps for infective juvenile (IJ) emergence and values are displayed as IJ per larva. Each dot represents one larva and crossbars show mean and standard deviation; no dot = not assessed. Mean colonization density and statistical evaluation are shown in Table S2 and data on larval survival in respective experiments can be found in Table S1

In a first experiment, we tested the effect of application timing for EPP-EPN combinations on LCW larvae. Survival curves based on pooled data of three repetitions (time-shift 1–3) are shown in Fig. 2A and data of individual repetitions in Table S1. Mortality was higher in all treatments compared to the control. Over all repetitions, the EPP-EPN combinations were significantly more lethal than EPP regardless of application timing and significantly different to EPN when EPN were added 6 h before EPP (Fig. 2A). In the individual repetitions, the simultaneous application of EPP and EPN reduced the mean survival time compared to single applications (EPN 6–7 h, EPP 9–20 h), and consistently resulted in a higher mortality (94–100%), whereas mortality of single applications was more variable (EPN 70–100%, EPP 50–80%; Table S1). Larva colonization by EPP 1 day after infection was not affected by the presence of EPN at any application time-point (Fig. 3A, 3C, Table S2). EPN reproduction (= emergence of infective juveniles) took place in the presence of EPP, but only in half of the larvae when EPN were added 6 h after EPP (Fig. 3B, 3D, Table S2).

In a second series of experiments, all three BCA were added simultaneously to LCW and BCB larvae. Survival curves based on pooled data (LCW 1–4 and BCB 1–4) are shown in Fig. 2B and C and data on individual repetitions of the experiments in Tables S3 and S4. In these experiments, EPF *M. brunneum* Bip5 and EPN *S. feltiae* RS5 alone were generally faster at killing larvae and caused higher mortality than EPP *P. chlororaphis* PCLRT03, which was significant for EPN and EPF in LCW and for EPF in BCB (Fig. 2B, C). The triple combination was the deadliest and fastest killing treatment against both insects and was significantly different to all single applications except for EPN in LCW. In individual repetitions, the triple combination caused 90–100% mortality in LCW and 80–95% in BCB (Tables S3, S4). In LCW, the EPP-EPN double combination was significantly more lethal compared to all other single and double applications (Fig. 2B). The EPN-EPF combination reached more consistently a high mortality compared to EPN and EPF, while the EPF-EPP combination behaved similarly to EPF (Table S3). In BCB, the EPN-EPF combination was the most lethal double combination, yet it was only significantly different to EPP (Fig. 2C). Both double combinations with EPP were only as lethal as the EPN or EPF partner alone, though significantly more effective than the EPP treatment.

Taken together, the triple combination was the most lethal treatment against both insect species. Faster and higher mortality was mainly caused by the combination of EPP with EPN for LCW and of EPN with EPF for BCB.

### Proliferation and Competition of BCA after Co-infection of *P. brassicae *and* D. balteata*

**Fig. 4:**
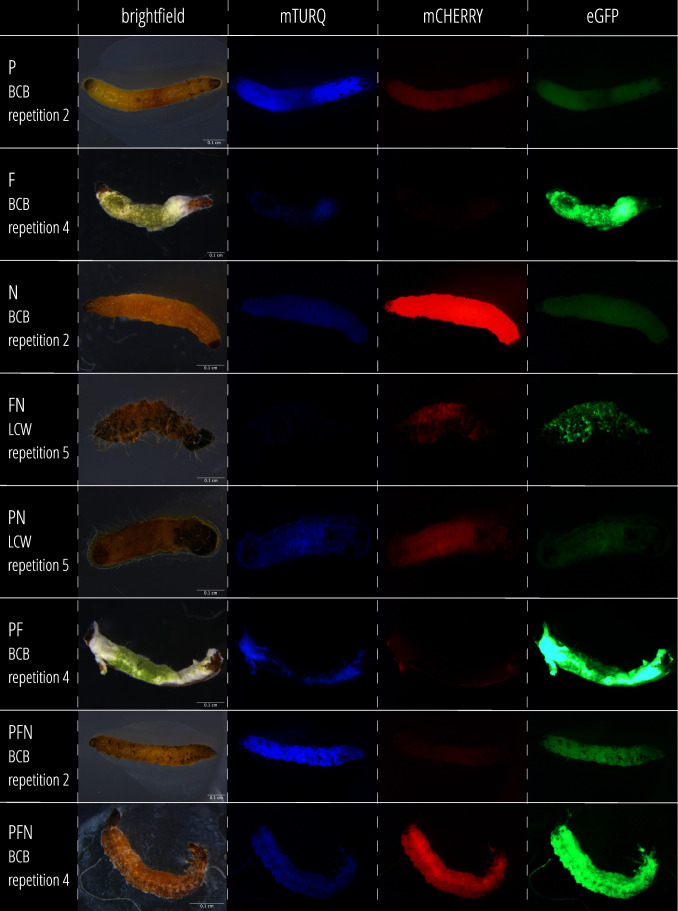
Pictures of *P. brassicae* (LCW) and *D. balteata* (BCB) larvae infected with EPP, EPN, and EPF under brightfield and fluorescence filters. Each row shows pictures of the same larva at 5 dpi acquired under a stereomicroscope using different filters: brightfield, ET CFP (mTURQ), ET mCHER (mCHERRY) and ET GFP (eGFP). The first column states the treatment and the experiment of the larva. Treatments: P = EPP *P. chlororaphis* PCLRT03-mturq, N = EPN *S. feltiae* RS5-mche, F = EPF *M. brunneum* Bip5-gfp, FN, PN, PF and PFN = double and triple applications of respective BCA. Information on fluorescence imaging is given in the Supplementary Methods

The development of the BCA populations inside their insect hosts was monitored in repetition 5 of the LCW and repetition 2 of the BCB experiment by selective plating (cfu/larva) and by qPCR (units/larva) and in addition pictures of dead larvae were taken with a fluorescence stereomicroscope (Fig. 1). In several pictures signals of two or even three fluorophores were detected, which indicated that BCA can co-exist in larvae at 5 dpi where we expected high BCA proliferation (Fig. 4).

**Fig. 5:**
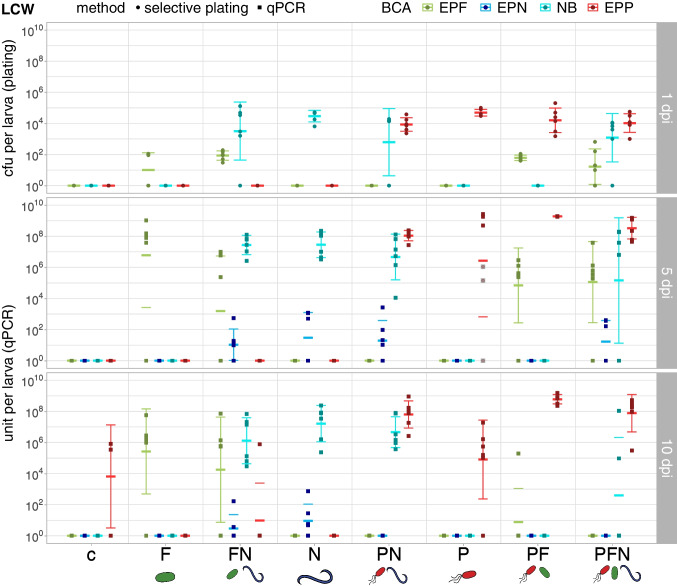
Colonization of *P. brassicae* (LCW) larvae by EPF, EPP, EPN and their associated NB. BCA counts in cfu and units per larva were assessed by selective plating (1 dpi) and qPCR (5 and 10 dpi) in LCW repetition 5. Treatments: c = control with no BCA application, P = EPP *P. chlororaphis* PCLRT03-mturq, N = EPN *S. feltiae* RS5-mche (associated with NB *X. bovienii* SM5-mcherry), F = EPF *M. brunneum* Bip5-gfp, FN, PN, PF and PFN = double and triple combinations of respective BCA. BCA: EPF = Bip5-gfp, EPN = RS5-mche, NB = SM5-mcherry, EPP = PCLRT03-mturq. At 1 dpi, six alive larvae, and at 5 and 10 dpi six dead larvae (control *n* = 3) were selected for homogenization. At 5 dpi, three alive larvae had to be taken in treatment P because not enough dead larvae were available. At 1 dpi, colonization was assessed by selective plating and values are displayed as colony forming units (cfu) per larva. At 5 and 10 dpi, colonization was assessed by qPCR and colonization values are displayed in units per larva (relative to bacteria cells, fungal spores and nematode IJ). Each dot or square represents one larva and crossbars show mean and standard deviation; outlined squares marked with a cross (in grey) indicate which larvae were still alive before homogenization at 5 dpi in treatment P. Mean colonization densities and statistical analyses are shown in Table S7, and the survival curves and corresponding data are displayed in Fig. S3 and Table S3

**Fig. 6:**
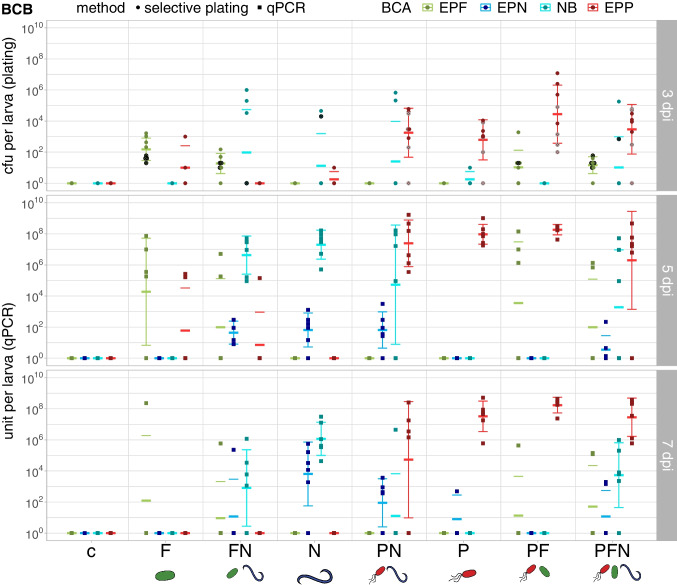
Colonization of *D. balteata* (BCB) larvae by EPF, EPP, EPN and their associated NB. BCA counts in cfu and units per larva were assessed by selective plating (3 dpi) and qPCR (5 and 7 dpi) in BCB repetition 2. Treatments: c = control with no BCA application, P = EPP *P. chlororaphis* PCLRT03-mturq, N = EPN *S. feltiae* RS5-mche (associated with NB *X. bovienii* SM5-mcherry), F = EPF *M. brunneum* Bip5-gfp, FN, PN, PF and PFN = double and triple combinations of respective BCA. BCA: EPF = Bip5-gfp, EPN = RS5-mche, NB = SM5-mcherry, EPP = PCLRT03-mturq. At 3 dpi, four alive and four dead larvae (control *n* = 4) and at 5 and 7 dpi six dead larvae (control *n* = 3) were selected for homogenization. At 3 dpi, colonization was assessed by selective plating and values are displayed as colony forming units (cfu) per larva. At 5 and 7 dpi, colonization was assessed by qPCR and colonization values are displayed in units per larva (relative to bacteria cells, fungal spores and nematode IJ). Each dot or square represents one larva and crossbars show mean and standard deviation; outlined dots marked with a cross (in black or grey) indicate that larvae were alive before homogenization at 3 dpi. Mean colonization density and statistical evaluation are shown in Table S8, and the survival curve and corresponding data are displayed in Fig. S4 and Table S4

EPP *P. chlororaphis* PCLRT03 were detected at 10^4^–10^5^ (LCW, 1 dpi) and 10^2^–10^5^ (BCB, 3 dpi) cfu/larva at the onset of the infection and population sizes reached 10^7^–10^9^ units/larva at later stages in cadavers (Figs. 5, 6, Tables S5, S6). Whereas EPP populations in BCB were not affected by co-inoculation with other BCA, mean EPP populations in LCW were elevated in cadavers at 10 dpi following applications of double and triple combinations.

EPF *M. brunneum* Bip5 was detected at 50–400 cfu/larva at 1 and 3 dpi and colonization levels in the single treatment were on average 10^7^–10^8^ units/larva at 5 dpi (Figs. 5 and 6, Tables S5, S6). EPF were clearly impacted by co-inoculation: in LCW, EPF colonization levels were lower in the combinations at 5 dpi and in BCB, EPF were detected less frequently in double and triple combinations at 3 dpi and 5 dpi.

EPN *S. feltiae* RS5 were present in both insects at 40–600 units/larva at 5 dpi but were detected in fewer larvae in the triple combination compared to the single EPN application (Figs. 5 and 6, Tables S5, S6). EPN populations in the single treatment increased on average 500-fold from 5 to 7 dpi in BCB, and decreased in both insects at 10 dpi. In the combination treatments, however, EPN were scarcely detected in BCB or LCW larvae at 7 and 10 dpi. Interestingly, in the EPP-EPN-combination at 7 dpi, half of the BCB larvae were occupied by EPN (400–800 units/larva) and EPP (10^6^–10^8^ units/larva), but not by nematode-associated bacteria (NB).

The NB *X. bovienii* SM5 was monitored additionally to the EPN. NB were detected in all LCW larvae in the EPN single treatment and in around half the larvae in combinations of EPN with other BCA at 1 dpi (Fig. 5) but only in a few dead BCB larvae at 3 dpi (Fig. 6). At 5 dpi, NB were present in almost all larvae on average at 10^7^ units/larva (Tables S5, S6). However, similar to EPN, NB were detected less frequently in the triple combination compared to all other EPN treatments, although not in lower numbers if present (Figs. 5, 6). In BCB, NB population size decreased with progressing cadaver decay from 5 to 7 dpi (Fig. 6, Table S6). In LCW, NB population size did not decrease from 5 to 10 dpi, yet NB disappeared in the triple treatment in two thirds of the larvae (Fig. 5, Table S5).

### Four Entomopathogens Can Co-exist Inside the Same Larva

**Fig. 7:**
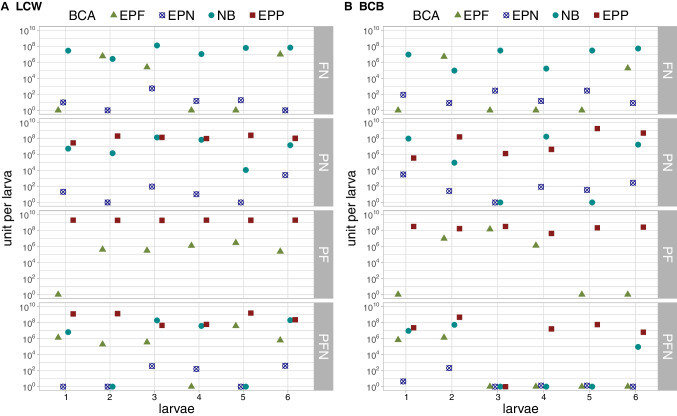
Colonization of individual *P. brassicae* (LCW) and *D. balteata* (BCB) larvae by EPF, EPP, EPN, and their associated NB. Colonization of six individual larvae by BCA after simultaneous application at 5 dpi in **A** LCW repetition 5 and **B** BCB repetition 2. BCA: EPF = *M. brunneum* Bip5-gfp, EPN = *S. feltiae* RS5-mche, NB = *X. bovienii* SM5-mcherry, EPP = *P. chlororaphis* PCLRT03-mturq. Treatments: FN = combined application of EPF and EPN (associated with NB), PN = EPP and EPN (with NB), PF = EPP and EPF, PFN = EPP, EPF and EPN (with NB). Colonization was assessed by qPCR and colonization values are displayed in units per larva (relative to bacteria cells, fungal spores, and nematode IJ). For improved readability, BCA that are not part of a double combination were removed in this plot; all datapoints are shown in Figs. 5 and 6

To further investigate co-existence, we looked at population sizes of the four entomopathogens inside six individual larvae for each combination treatment at 5 dpi when larvae had died, but cadavers were not yet decayed (Fig. 7). In the EPF-EPN combination, NB were present in all BCB and LCW larvae together with either EPN or EPF, except for one LCW and two BCB larvae where all three organisms were detected. EPF were present in five out of twelve larvae. In the EPP-EPN combination, both bacteria colonized nearly all larvae and EPN were also found in the majority of the larvae. In the EPP-EPF combination, EPP were always present in high numbers whereas EPF had propagated in most LCW larvae but only half of the BCB. In the triple combination, EPP colonized all larvae at high population sizes, except for one BCB larva that was not colonized by any BCA. In BCB, all four entomopathogens were present in two larvae, EPP alone in two and both bacteria in one. In LCW, two larvae were colonized by all four entomopathogens, two by EPP and EPF, and two by both bacteria and either EPN or EPF. The co-colonization of individual larvae by multiple BCA was also observed by fluorescence microscopy (Fig. 4).

In summary, the single larva analysis revealed that EPP and NB can co-colonize insect larvae together with EPN or EPF. EPF are mostly not impacted by EPP, but inhibited in co-infections with EPN. The application of the triple combination leads in most cases to the final establishment of only two entomopathogens with EPP always among these. Yet, in spite of the observed exclusion, in some cases, all four organisms can co-exist and grow together in the same cadaver (Figs. 4, 7).

## Discussion

In this study, we investigated the impact of a BCA consortium on larval mortality, killing speed, and BCA proliferation in two taxonomically distant insect pests living in different habitats. As we predicted, BCA combinations were generally more deadly and faster in killing larvae than single applications and the triple combination was the most lethal treatment in both insects. In the BCB experiments, the results of the repetitions 1, 3, and 4 have to be considered with care since the mortality in the control was high because experimental conditions were unfavorable for the animals. Still, the same tendencies as for repetition 2 were observed, i.e., faster killing and higher mortality in the triple combination compared to single applications. In previous greenhouse and field trials, the same consortium decreased insect survival and damage on plants attacked by a Dipteran pest, the cabbage maggot *D. radicum*, by 50% [27]. In previous laboratory assays, the EPP were the most effective agents when applied alone, and double combinations of EPP with either EPN or EPF had synergistic effects [27]. In this study, EPF and EPN were more effective than EPP in single applications. The best double combinations were EPN-EPP against the Lepidopteran LCW and EPN-EPF against the Coleopteran BCB. These findings indicate that the performance of the BCA and synergisms between individual consortium members vary depending on the host insect. Despite the variability in efficacy observed for single and double applications, the triple combination was effective against all three insect pests targeted across our two studies. Similar results of BCA combinations have been observed in other studies. For example, Jabbour et al. [36] found a linear increase in mortality with increasing pathogen species richness when infecting Colorado potato beetles with combinations of three EPN (*Heterorhabditis megidis*, *S. feltiae*, *S. carpocapsae*) and one EPF (*Beauveria bassiana*). The combination of one EPN with EPF had the highest impact on mortality and resulted in synergistic effects. Bueno-Pallero et al. [24] used different inoculation methods for EPF (*B. bassiana*) which affected insect mortality yet combinations of EPF and EPN (*S. feltiae*) additively increased mortality in nearly all settings.

During the infection, the BCA have to overcome the insect immune defense and compete with the insect microflora or scavengers [37–39]. The three BCA have different infection pathways: EPN enter through natural openings and release the NB into the hemolymph, EPF penetrate the cuticle and EPP need to be ingested [15, 18, 22]. Furthermore, the four entomopathogens (the three BCA and the nematode-associated NB) all produce a cocktail of insecticidal and antimicrobial toxins [23, 40, 41]. EPF and NB both produce compounds that modulate and suppress the insect immune response [42, 43]. We assume that the different infection pathways and modes of action of the consortium members contribute to the overall activity of the consortium against different insect pests. An insect is more likely to succumb to infection and might do so faster when challenged with different physical damages and a larger variety of toxic compounds. Moreover, it is highly unlikely that an insect develops resistance against all three BCA simultaneously, especially since our colonization data suggest that all are involved in the infection at those stages which are relevant for resistance development. Potentially, our consortium of three potent BCA has a greater range of target species than the single BCA or even the double combinations. This indicates that it may be applied against various agronomic pests from the families Lepidoptera, Coleoptera, and Diptera.

We further hypothesized that the BCA might hinder each other’s proliferation in the cadaver due to competition for nutrients or antimicrobial interactions. Studies showing that both bacteria and the fungus inhibit each other in vitro[25, 27, 44, 45] indicate that the susceptibility towards the opponent’s antimicrobials is given. Thus, one of our major aims was to co-monitor all organisms after simultaneous host attack. To the best of our knowledge, our study is the first to observe the co-occurrence of four entomopathogens associated with biocontrol during the course of an infection and provides novel insights to understanding their interactions within the host. The proliferation of EPF and EPN is clearly affected by the presence of other BCA, especially in the triple combination, while EPP proliferate equally well or even better in combinations compared to single applications. Possibly, EPP profit in co-infections from EPF or EPN entering the insect and damaging the tissue. EPP could then reach the hemolymph more easily where they can multiply and reach high numbers. In triple combinations, EPP always prevailed after 5 days while one, two, or even three of the other entomopathogens had vanished in most larvae (Fig. 7). It is remarkable that EPP colonize insects to such high densities, since insects were only recently discovered as an ecological niche of EPP [33]. Even though EPP are highly competitive in the rhizosphere [13–15], they do not seem to outcompete the other entomopathogens in double combinations neither during infection nor during colonization of the cadaver. EPN could reproduce in larvae co-infected with EPP and IJ emergence was not reduced in infections with simultaneous application (Fig. 3). Blanco-Pérez et al. [38] observed that high competition in the cadaver affected IJ fitness, but this was not assessed in our study. Ogier et al. [25] discovered EPP in the “frequently associated microbiome” of *Steinernema* IJ from lab and natural environments, suggesting a close link between EPP and EPN. In several BCB cadavers, only EPP and EPN but no NB were detected (Fig. 6). Possibly, IJ carrying few NB had formed at this time-point and NB were below detection limit, or EPN could reproduce without the presence of NB.

In comparison to what we observe with EPP, EPF and EPN were unable to proliferate in the same cadaver for a long time. Tarasco et al. [44] observed a strong competition for space and nutrients between EPF and EPN. EPF and EPN spread from their primary infection site and usually one outcompeted the other, though in some cases, both EPF and EPN symptoms were observed on different parts of individual cadavers. In our study, EPF and NB were able to co-colonize cadavers at relatively high densities, yet EPF and EPN were rarely detected in the same cadaver. Probably, once EPF have established, they presumably suppress EPN reproduction but not that of their symbionts.

Interestingly, a third of the cadavers were colonized by all four BCA at 5 dpi (Fig. 7). The entomopathogens seem to be sufficiently tolerant to each other’s antimicrobial substances to proliferate in the same cadaver, and competition for resources might be more limiting for co-colonization than direct antimicrobial interactions. We assume that the competition inside the cadaver, i.e., inhibition of EPF sporulation and EPN reproduction, does most likely not lower biocontrol efficacy itself, at least in inundative biocontrol approaches. Biopesticide strategies are mainly based on repeated BCA treatments at intervals depending on field persistence of BCA and the pest pressure and do not rely on the performance of subsequent generations of the BCA.

In conclusion, the combination of three BCA might increase biocontrol efficacy. The co-infections resulted in increased killing speed and mortality against two agricultural insect pests. When comparing the two insect species, different BCA double combinations showed similar colonization dynamics but distinct insect killing effects. The competition between the entomopathogens increased with advancing decay of the cadaver and limitation of nutrients, and EPP finally dominated the cadaver in all combinations. Our findings indicate that the studied entomopathogenic pseudomonads, nematodes including their symbionts and fungi are compatible, can jointly infect insect larvae, and can potentially be used to control a range of insect pests.

## Supplementary Information

Below is the link to the electronic supplementary material.

Supplementary file1 (DOCX 761 KB)

## Data Availability

The data generated during the current study are available from the corresponding authors on reasonable request.

## Abbreviations

BCA: Biocontrol agent(s)
EPP: Entomopathogenic pseudomonads
EPF: Entomopathogenic fungi
EPN: Entomopathogenic nematodes
NB: Nematode-associated bacteria
LCW: Large cabbage white
BCB: Banded cucumber beetle
cfu: Colony-forming units
IJ: Infective juveniles
