# Supplementary material for: When Competitors Join Forces: Consortia of Entomopathogenic Microorganisms Increase Killing Speed and Mortality in Leaf- and Root-Feeding Insect Hosts
Source: Microb Ecol. 2023 Feb 27;86(3):1947–60. doi: 10.1007/s00248-023-02191-0 (PMC10497674; doi:10.1007/s00248-023-02191-0)
Supplement: Supplementary file 1 — Supplementary file1 (DOCX 761 KB) [file 248_2023_2191_MOESM1_ESM.docx]

# When competitors join forces: Consortia of entomopathogenic microorganisms increase killing speed and mortality in leaf- and root-feeding insect hosts

Anna Spescha^1,†^, Maria Zwyssig^1,†^, Mathias Hess Hermida^1,2^, Aurélie Moix^1^, Pamela Bruno^3^, Jürg Enkerli^4^, Raquel Campos-Herrera^5^, Giselher Grabenweger^2^, Monika Maurhofer^1^

Affiliations:

1 Institute of Integrative Biology, ETH Zurich, Switzerland

2 Research Group Extension Arable Crops, Agroscope, Zurich, Switzerland

3 Division of Agricultural Entomology, Department of Crop Sciences, Georg-August-Universität Göttingen, Germany

4 Research Group Molecular Ecology, Agroscope, Zurich, Switzerland

5 Instituto de Ciencias de la Vid y del Vino (ICVV), CSIC, Gobierno de La Rioja, Universidad de La Rioja, Logroño, Spain

† contributed equally to this study

Corresponding authors: Monika Maurhofer, monika.maurhofer@usys.ethz.ch and
Anna Spescha, anna.spescha@usys.ethz.ch

# Supplementary Information

Content

- Supplementary Material and Methods
  - Insect Rearing
  - Reassociation of *Steinernema feltiae* with modified *Xenorhabdus bovienii*
  - Determining larval colonisation with BCA
- Supplementary Results
  - Supplementary Tables
  - Supplementary Figures

## Supplementary Material and Methods

### Insect Rearing

Eggs of the large cabbage white (LCW) *Pieris brassicae* (Lepidoptera: Pieridae) were obtained from the Biocommunication Group (Institute of Agricultural Sciences, ETH Zurich, Switzerland) and incubated in petri dishes (ø 150 mm) with Savoy cabbage (*Brassica oleracea* var. *sabauda* L.) leaves. Freshly hatched larvae were transferred into 720 ml BugDorms (BugDorm, Taiwan) and fed with Savoy cabbage. In the evening before experiments started, sufficient larvae, mainly early 3^rd^ instar, were collected in petri dishes (ø 150 mm) with a moistened filter paper (ø 150 mm, Conatex, Germany) and a Savoy cabbage leaf. In the morning before an experiment started, larvae were starved for approx. 6 h. Eggs and larvae were incubated during their entire lifespan in a phytotron with 60% rH and a day-night cycle with 16 h at 25°C and 12 kLux followed by 8 h at 20°C and darkness. Savoy and Chinese cabbage used for feeding was bought at supermarkets (Coop or Spar, Switzerland).

Eggs of the banded cucumber beetle (BCB) *Diabrotica balteata* LeConte (Coleoptera: Chrysomelidae) were provided by Syngenta Crop Protection (Stein, Switzerland). Eggs were stored in ø 150 mm petri dishes with a moistened filter paper (ø 150 mm) until hatching. For larval rearing, plastic containers (24 x 16 x 10 cm, Topline, Migros, Switzerland) were prepared by removing a rectangle from the lid and sealing it with a 125 µm mesh (03-125/45, Sefar, Switzerland). Freshly hatched larvae were transferred into these rearing boxes and covered with 5-day-old germinated maize seedlings (*Zea mays mays* variety Damaun KS, sativa, Switzerland) and peat substrate (Jiffy Products International, Moerdijk, the Netherlands). In the morning of an experiment, sufficient larvae, mainly early 2^nd^ instar (approximately one week after hatching), were collected in petri dishes (ø 150 mm) with a moistened filter paper (ø 150 mm) and starved for 6 h. Eggs and larvae were incubated in the dark at 27°C for rearing and at 25°C with 70% relative humidity during experiments.

### Strain selection and inoculation concentrations

In our previous study [1], we have selected the biocontrol agents EPP *P. chlororaphis* PCLRT03, EPN *S. feltiae* RS5 and EPF *M. brunneum* Bip5 based on their efficacy against the cabbage maggot *Delia radicum* and their compatibility. In greenhouse, semi-field and field experiments, soil (EPP, EPN, EPF) and root (EPP, EPF) colonisation densities were not affected by the presence of one or both other BCA. Furthermore, synergistic effects were observed when applying EPP with EPN or EPF in laboratory assays.

To further study the interaction between these three BCA, the laboratory assays with LCW and BCB were developed. Different inoculation concentrations and methods were tested in pre-experiments (data not shown). Similar to the previous study, the food was submerged in a high-concentration bacteria suspension prior to feeding. A higher concentration was used for BCB than for LCW (5 resp. 2.5 x 10^8^ cfu/ml) due to the lower surface area of maize seedlings compared to Chinese cabbage. For EPN, it was determined that 50 IJ per larva are sufficient to ensure a successful infection while observing little variation between wells. For EPF, relatively high concentrations were used for both insects to ensure that the spores get in contact with the larvae: LCW were submerged 5 s in 10^7^ spores/ml, while BCB were drenched with 5 x 10^6^ spores. Due to their lower size, BCB larvae were more fragile in handling and were drenched with the EPF suspension rather than immersed in it.

### Reassociation of *Steinernema feltiae* with modified *Xenorhabdus* *bovienii*

*Xenorhabdus bovienii* SM5-mcherry was stored in 20% glycerol at -80°C and grown on Lysogeny broth (LB) agar supplemented with ampicillin 40 mg/l and kanamycin 50 mg/l for two days at 28°C. LB liquid cultures were incubated over night at 28°C and 180 rpm. Subsequently, 6^th^-instar *Galleria mellonella* larvae (Hebeisen fisher store, Zurich, Switzerland) were injected with 10 µl of a 1:1 mix of kanamycin (100 mg/ml) and SM5 overnight culture following the injection protocol described in Flury et al. [2]. The larvae were transferred to a petri dish (ø 60 mm) containing a filter paper (ø 55 mm, Conatex, Germany) and 400 µl *Steinernema* *feltiae* RS5 wildtype (1000 IJ/ml) were added onto the paper. Cadavers and freshly emerging nematodes were examined under a fluorescence microscope (Leica DM2500, Leica Microsystems, Germany) and a fluorescence stereomicroscope (LEICA M205FCA, Leica Microsystems, Germany) for an mcherry signal (Fig. A1). The emerging population was collected using the White-Trap method [3] and called RS5-mche. To uphold selection pressure, *G. mellonella* were injected with 10 µl kanamycin (10 mg/ml) before infection with RS5-mche for further multiplication. Based on stereomicroscope observations, we estimated that >90% of RS5-mche IJ carry SM5-mcherry bacteria. To test the infectivity of the new population, *P. brassicae* larvae were infected with RS5-mche or RS5 wildtype and the survival of 18 larvae per strain was monitored as described in the main text. The survival was very similar and statistically not significantly different for RS5-mche and RS5 wildtype (Fig. A2).

| 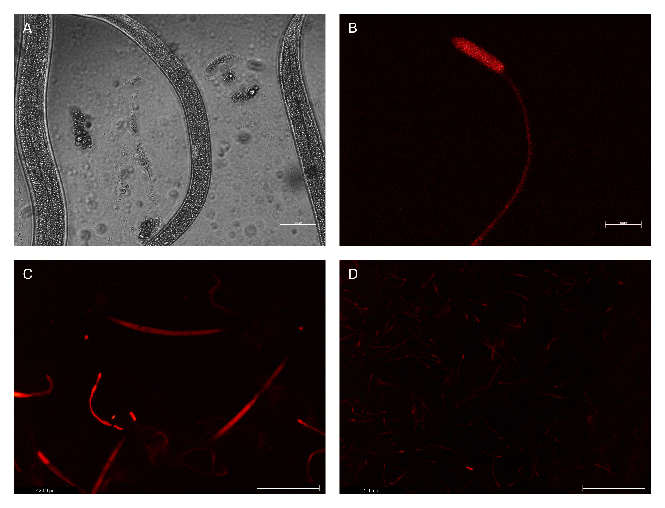 |
| --- |
| **Fig. A1** *Xenorhabdus* sp. SM5-mcherry re-associated with *Steinernema feltiae* RS5.  Pictures A and B were taken using a microscope and show the same IJs, A with brightfield and B with an mcherry-filter. Pictures C and D were taken using a stereomicroscope and an mcherry-filter. Pictures were taken three days after IJ started to emerge from *G. mellonella* cadavers. Scale bars represent 40.56 µm in A and B, 428.3 µm in C and 1.4 mm in D. |

|  |
| --- |
| **Fig. A2** Survival of *P. brassicae* (LCW) larvae after infection with EPN  The figure shows the larval survival of *P. brassicae* to compare the infectivity of *S. feltiae* RS5 wildtype (WT) and RS5-mche. No significant differences (*P* < 0.05) were detected according to a log-rank test and a pairwise survival difference. |

## Determining larval colonisation with BCA

Larvae were surface disinfected by submerging them for 20 s each, first in 70% (v/v) EtOH, subsequently in 0.05% (w/v) SDS, 70% (v/v) EtOH and finally in ddH_2_O before homogenization.

### Selective Plating

Two different homogenization protocols were applied. In EPP x EPN time-shift experiments, larvae were homogenized in 2 ml Eppendorf tubes containing 1 ml 0.9% NaCl with the Polytron RT-MR2100 blender, 500W (Kinematica, Switzerland) as described by Flury et al. [4]. For each one LCW (repetition 5) and BCB (repetition 2) experiment, larvae were disrupted in 2 ml Eppendorf tubes containing 100 µl 0.9% NaCl using one sterilized ø 5 mm bead and the MM300 TissueLyser (Retsch, Germany) for 2 x 45 s at 30/s. After bead disruption, 900 µl 0.9% NaCl was added to each tube. Larval homogenates were plated on King’s B agar supplemented with cycloheximide 100 mg/l, chloramphenicol 13 mg/l and gentamycin 10 mg/l (KB^++G^) to detect EPP, and in LCW and BCB experiments additionally on selective medium (SM) agar to detect EPF and on LB agar supplemented with ampicillin 40 mg/l and kanamycin 50 mg/l (LB^AK^) to detect NB. For treatments containing EPF, for each larva 100 µl of the undiluted homogenate was plated. For treatments containing EPP or EPN, 10 µl droplets of 10-fold serial dilutions (10^0^ – 10^-5^) were spotted on the respective medium per larva. To check for cross-contaminations, 100 µl of the undiluted homogenate was plated on all the media using Drigalski spatula for half of the extracted larvae. The detection limits were 100 cfu/larva for EPP and NB and 10 cfu/larva for EPF.

### qPCR

At 3 dpi, 600 µl of larval homogenate was pelleted in 1.5 ml Eppendorf tubes centrifuged at 3500 rcf for 30 s and the pellet was frozen at -20°C. For later time points, dead LCW and BCB larvae were collected at 2 dpi and at 4 dpi, respectively, surface disinfected, transferred into 2 ml tubes containing 10 µl 0.9% NaCl and further incubated under experimental conditions. At 5, 7 and 10 dpi, previously surface disinfected larvae (n=6, control n=3) were frozen at -20°C. After thawing, beads were added and larvae were disrupted with the TissueLyser as described above. DNA was extracted using the QIAGEN Blood & Tissue kit according to the manufacturer’s instructions with the adaptions for insect samples regarding sample lysis. The samples were lysed by adding 180 µl buffer ATL and 20 µl proteinase K and incubated at 56°C and 300 rpm overnight (approx. 16 h) for larvae collected at 5, 7 and 10 dpi and for 4 h for pellets collected at 3 dpi. For DNA elution, 100 µl elution buffer was used. DNA concentration was measured using NanoDrop2000 (Thermo Fisher Scientific, MA, USA). qPCR was performed using EvaGreen®, a Lightcycler® 480 (Roche, Switzerland) and a Mosquito® HV pipetting robot (SPT Labtech, UK) at the Genetic Diversity Center (GDC, Zurich, Switzerland). For the master mix, 2.375 µl miliQ, 0.25 µl BSA, each 0.25 µl forward and reverse primer (10 nM) and 2 µl EvaGreen® 5x were mixed per sample, and 1 µl undiluted sample DNA added. Samples were run in duplicates with 12 min initial activation at 95°C, 35 cycles of 15 s denaturation at 95°C, 30 s annealing at 60°C, 30 s extension at 72°C, and a stepwise melting curve with 15 s at 95°C, 1 min at 55°C and a continuous increase to 95°C. Published species-specific primers were used for *S. feltiae* (Campos-Herrera et al. [5]) and *M. brunneum* (Ma1763 and Ma2079, Schneider et al. [6]) and primers targeting the *phzF* gene involved in phenazine biosynthesis for *P. chlororaphis* (Imperiali et al. [7]). Primers targeting the *rpoD* gene in *X. bovienii* SM5 were designed and kindly provided by Tabea Patt (ETH Zurich) (SM5_F: TTT CAC CGC TAC ACG TGG AAT, SM5_R: AGC GTA AAT AGC GCT GTT GAT TGA). For separate standard curves, DNA of 300 IJ RS5, 10^8^ spores Bip5, 10^9^ cells SM5-mcherry and PCLRT03-mturq was extracted and 10-fold dilution series with 5 steps prepared. Colonisation values relative to cells, spores and IJ were calculated and displayed as unit per larva. These values need to be interpreted with caution because EPN and EPF are not in the same state in the larvae as in the standard curve, i.e. EPN reproduce in larvae and are present in juvenile, adult and egg stages, while EPF grow mainly as hyphae inside the cadaver and only form spores when the larva is overgrown with mycelium. The qPCR data was cut-off at a Cp value of 28, resulting in approximate detection limits (per larva) of 1 IJ for *S. feltiae*, 10^5^ spores for *M. brunneum*, 10^4^ cells for *X. bovienii* and 10^5^ cells for *P. chlororaphis*, with small variations between qPCR runs.

### White trap

In EPP x EPN time-shift experiments, freshly deceased LCW larvae were transferred on a filter paper (ø 30 mm, Whatman, Huberlab, Switzerland) on the lid of a ø 30 mm petri dish within a ø 60 mm petri dish filled with approx. 8 ml tap water. After four weeks at 22°C in the dark, the emerged IJ in the tap water were counted under a stereomicroscope and IJ emergence per larva was calculated.

### Fluorescence stereomicroscopy

LCW larvae were transferred from 6-well into 12-well plates and BCB larvae from 12-well into 24-well (CELLSTAR®, Greiner Bio-One, Austria) plates. Pictures were acquired using a LEICA M205FCA stereomicroscope equipped with a Leica DFC 7000T CCD colour camera and the software Leica ApplicationSuite X (Leica Microsystems). Serial images were captured in the brightfield as well as using the filters ET CFP (10447409, A: 436/20 E: 480/40), ET mCHER (10450195, 560/40 E: 630/75) and ET GFP (10447408, A: 470/40 E: 525/50). For the brightfield conditions, 40 s exposure, 2x gain and 20% lamp intensity were used whereas for the fluorescence filters, 10x gain and no lamp was used, with 950 s exposure for LCW and 400 s for BCB experiments. Images were processed using the Fiji package of ImageJ (https://fiji.sc).

## References

1. Spescha A, Weibel J, Wyser L, et al (2023) Combining entomopathogenic *Pseudomonas* bacteria, nematodes and fungi for biocontrol of a soil-borne insect pest. Agric Ecosyst Environ, in revision.

2. Flury P, Vesga P, Péchy-Tarr M, et al (2017) Antimicrobial and insecticidal: Cyclic lipopeptides and hydrogen cyanide produced by plant-beneficial *Pseudomonas* strains CHA0, CMR12a, and PCL1391 contribute to insect killing. Front Microbiol 8:. https://doi.org/10.3389/fmicb.2017.00100

3. Campos-Herrera R, Jaffuel G, Chiriboga X, et al (2015) Traditional and molecular detection methods reveal intense interguild competition and other multitrophic interactions associated with native entomopathogenic nematodes in Swiss tillage soils. Plant Soil 389:237–255. https://doi.org/10.1007/s11104-014-2358-4

4. Flury P, Vesga P, Dominguez-Ferreras A, et al (2019) Persistence of root-colonizing *Pseudomonas protegens* in herbivorous insects throughout different developmental stages and dispersal to new host plants. ISME J 13:860–872. https://doi.org/10.1038/s41396-018-0317-4

5. Campos-Herrera R, El-Borai FE, Stuart RJ, et al (2011) Entomopathogenic nematodes, phoretic *Paenibacillus* spp., and the use of real time quantitative PCR to explore soil food webs in Florida citrus groves. J Invertebr Pathol 108:30–39. https://doi.org/10.1016/j.jip.2011.06.005

6. Schneider S, Widmer F, Jacot K, et al (2012) Spatial distribution of *Metarhizium* clade 1 in agricultural landscapes with arable land and different semi-natural habitats. Appl Soil Ecol 52:20–28. https://doi.org/10.1016/J.APSOIL.2011.10.007

7. Imperiali N, Dennert F, Schneider J, et al (2017) Relationships between Root Pathogen Resistance, Abundance and Expression of *Pseudomonas* Antimicrobial Genes, and Soil Properties in Representative Swiss Agricultural Soils. Front Plant Sci 8:. https://doi.org/10.3389/fpls.2017.00427

## Supplementary Results

## Supplementary Tables

| **Table S1**  Effect of EPP and EPN time-shift application on *P. brassicae* (LCW) larvae | | | | | | | | | |
| --- | --- | --- | --- | --- | --- | --- | --- | --- | --- |
| Treat | Repetition 1 | | | Repetition 2 | | | Repetition 3 | | |
|  | mean | mort. | stats | mean | mort. | stats | mean | mort. | stats |
| c | 71.9 | 31.2 | a | 68.4 | 13.6 | a | 72.5 | 13.6 | a |
| N t0 | 49.9 | 81.2 | bcd | 47.5 | 68.2 | bcd | 40.2 | 100.0 | b |
| N t-6h | NA | NA | NA | 53.8 | 71.4 | bc | 37.0 | 83.3 | b |
| P | 64.9 | 50.0 | ab | 60.9 | 61.1 | b | 43.9 | 83.3 | b |
| PN t-6h | 36.4 | 91.7 | cd | 34.8 | 88.8 | d | 33.7 | 94.1 | b |
| PN t0 | 44.1 | 100.0 | d | 40.4 | 94.4 | cd | 34.8 | 100.0 | b |
| PN t+6h | 59.2 | 83.3 | bc | 35.5 | 94.1 | d | 34.0 | 100.0 | b |
| Results from the EPP x EPN time-shift experiment. Mean = mean survival time (in hours). Mort. = final larval mortality (in percent) at the end of the experiment with n = 18 larvae per treatment. Stats = pairwise comparison of survival curves; different letters indicate statistically significant differences at *P* < 0.05. Treatments: c = control with no BCA application, N t0 = EPN *S. feltiae* RS5, N t-6h = EPN applied 6 h earlier, P = EPP *P. chlororaphis* PCLRT03-gfp, PN t-6h = EPN applied 6 h before EPP, PN t0 = EPN and EPP applied simultaneously, PN t+6h = EPN applied 6 h after EPP. | | | | | | | | | |

| **Table S2** Proliferation of EPP and EPN in *P. brassicae* (LCW) larvae in the time shift experiment | | | | | | | |
| --- | --- | --- | --- | --- | --- | --- | --- |
| Treat | control | N t0 | N t-6h | P | PN t-6h | PN t0 | PN t+6h |
| Repetition 2 | | | | | | | |
| EPP | 9.0 x 10^2^ | 4.5 x 10^1^ | ND | 2.6 x 10^7^ | 1.9 x 10^6^ | 3.0 x 10^5^ | 1.0 x 10^6^ |
| EPN | ND | 7.2 x 10^3^ | 2.3 x 10^4^ | ND | 5.0 x 10^3^ | 6.3 x 10^3^ | 4.9 x 10^3^ |
| Repetition 3 | | | | | | | |
| EPP | 2.5 x 10^1^ | BD | ND | 4.4 x 10^6^ | 7.7 x 10^6^ | 3.5 x 10^6^ | 3.2 x 10^5^ |
| EPN | ND | 8.2 x 10^2^ | 3.2 x 10^3^ | ND | 1.4 x 10^4^ | 1.1 x 10^4^ | 3.1 x 10^2^ |
| Data shown are mean colonisation by EPP *P. chlororaphis* PCLRT03-gfp in cfu per larva as determined at 1 dpi by selective plating (n = 6) and mean proliferation of EPN *S. feltiae* RS5 as emerging IJ per larva as determined with white traps (n = 6), respectively. Treatments: c = control with no BCA application, N t0 = EPN *S. feltiae* RS5, N t-6h = EPN applied 6 h earlier, P = EPP *P. chlororaphis* PCLRT03-gfp, PN t-6h = EPN applied 6 h before EPP, PN t0 = EPN and EPP applied simultaneously, PN t+6h = EPN applied 6 h after EPP. BD = below detection; ND = not determined. No significant differences among treatments (*P* < 0.05) were detected according to an Anova and TukeyHSD test. | | | | | | | |

| **Table S3** Effect of EPP, EPF and EPN applied alone and in combinations on *P. brassicae* (LCW) larvae | | | | | | | | | | | | | | | |
| --- | --- | --- | --- | --- | --- | --- | --- | --- | --- | --- | --- | --- | --- | --- | --- |
| Treatment | Repetition 1 | | | Repetition 2 | | | Repetition 3 | | | Repetition 4 | | | Repetition 5 | | |
|  | mean | mort. | stats | mean | mort. | stats | mean | mort. | stats | mean | mort. | stats | mean | mort. | stats |
| control | 75.2 | 18.8 | a | 59.2 | 37.5 | a | 73.3 | 5.9 | a | 76.8 | 0.0 | a | 114.0 | 3.7 | a |
| F | 64.1 | 93.8 | c | 62.7 | 56.3 | a | 47.6 | 82.3 | cd | 43.2 | 100.0 | d | 50.0 | 100.0 | c |
| N | 55.4 | 76.5 | bc | 44.6 | 93.8 | bc | 35.7 | 94.4 | de | 63.8 | 50.0 | b | 34.8 | 95.8 | b |
| P | 66.9 | 40.0 | a | 53.1 | 52.9 | ab | 68.5 | 41.2 | b | 54.5 | 77.8 | bc | 111.0 | 20.8 | a |
| FN | 52.9 | 100.0 | b | 46 | 75.0 | abc | 37.0 | 94.4 | de | 46.6 | 100.0 | bc | 31.8 | 100.0 | b |
| PN | 36.2 | 100.0 | d | 37.8 | 100.0 | c | 32.4 | 100.0 | e | 52.3 | 73.7 | bcd | 33.9 | 95.8 | b |
| PF | 59.1 | 92.3 | bc | 64.1 | 58.8 | a | 51.0 | 83.3 | c | 47.2 | 100.0 | cd | 42.8 | 100.0 | c |
| PFN | 39.0 | 100.0 | d | 41.9 | 94.1 | bc | 32.4 | 100.0 | e | 46.9 | 89.5 | cd | 35.3 | 100.0 | b |
| Data shown are results from LCW repetition 1-5 with single and combined simultaneous application of EPP, EPF and EPN. Mean = mean survival time (in h). Mort. = final larval mortality (in percent) at the end of an experiment with n = 18 larvae per treatment. Stats = pairwise comparison of survival curves; different letters indicate statistically significant differences at *P* < 0.05. Treatments: control = no BCA application, P = EPP *P. chlororaphis* PCLRT03-gfp or PCLRT03-mturq (repetition 5), N = EPN *S. feltiae* RS5 or RS5-mche (repetition 5), F = EPF *M. brunneum* Bip5 or Bip5-gfp (repetition 5), FN, PN, PF and PFN = double and triple combinations of respective BCA. | | | | | | | | | | | | | | | |

| **Table S4** Effect of EPP, EPF and EPN applied alone and in combinations on *D. balteata* (BCB) larvae | | | | | | | | | | | | |
| --- | --- | --- | --- | --- | --- | --- | --- | --- | --- | --- | --- | --- |
| Treat | Repetition 1 | | | Repetition 2 | | | Repetition 3 | | | Repetition 4 | | |
|  | mean | mort. | stats | mean | mort. | stats | mean | mort. | stats | mean | mort. | stats |
| control | 3.5 | 65.9 | ab | 5.4 | 41.5 | a | 4.7 | 65.6 | ab | 4.7 | 68.7 | a |
| F | 2.7 | 87.2 | bc | 3.7 | 83.6 | cd | 4.8 | 52.4 | a | 3.9 | 80.9 | ab |
| N | 2.9 | 92.5 | bc | 4.3 | 70.8 | bc | 4.7 | 70.7 | ab | 4.2 | 76.9 | ab |
| P | 4.0 | 65.9 | a | 4.8 | 62.9 | b | 4.8 | 68.9 | a | 4.3 | 73.9 | ab |
| FN | 3.3 | 94.9 | bc | 3.5 | 90.3 | d | 4.5 | 78.3 | ab | 3.8 | 84.9 | b |
| PN | 3.0 | 87.8 | bc | 4.7 | 74.6 | b | 4.6 | 70.8 | ab | 4.4 | 73.9 | ab |
| PF | 2.9 | 82.9 | abc | 4.4 | 95.2 | c | 4.7 | 69.2 | ab | 4.4 | 72.3 | ab |
| PFN | 2.2 | 97.3 | c | 3.7 | 95.2 | d | 4.2 | 87.7 | b | 4.0 | 80.9 | ab |
| Data shown are results from BCB repetition 1-4 with simultaneous EPP, EPF and EPN single and combined applications. Mean = mean survival time (in days). Mort = final larval mortality (in percent) at the end of an experiment with n = 60 larvae per treatment. Stats = pairwise comparison of survival curves; different letters indicate statistically significant differences at *P* < 0.05. Treatments: control = no BCA application, P = EPP *P. chlororaphis* PCLRT03-mturq, N = EPN *S. feltiae* RS5-mche, F = EPF *M. brunneum* Bip5-gfp, FN, PN, PF and PFN = double and triple application of respective BCA. | | | | | | | | | | | | |

| **Table S5** Colonisation of *P. brassicae* (LCW) larvae by EPF, EPN, NB and EPP | | | | | | | | |
| --- | --- | --- | --- | --- | --- | --- | --- | --- |
| Treat | control | F | N | P | FN | PN | PF | PFN |
| 1 dpi, selective plating | | | | | | | | |
| EPF | BD | 5.2 x 10^1^ | BD | BD | 1.0 x 10^2^ | 6.5 x 10^1^ | BD | 1.4 x 10^2^ |
| NB | BD | BD | 3.6 x 10^4^ | BD | 3.6 x 10^4^ | BD | 1.0 x 10^4^ | 5.5 x 10^3^ |
| EPP | BD | BD | BD | 5.4 x 10^4^ | BD | 4.9 x 10^4^ | 1.3 x 10^4^ | 1.9 x 10^4^ |
| 5 dpi, qPCR | | | | | | | | |
| EPF | BD | 2.4 x 10^8^ | BD | BD | 2.7 x 10^6^ | BD | 8.5 x 10^5^ | 6.8 x 10^6^ |
| EPN | BD | BD | 4.8 x 10^2^ | BD | 9.8 x 10^1^ | 4.6 x 10^2^ | BD | 1.6 x 10^2^ |
| NB | BD | BD | 8.3 x 10^7^ | BD | 5.0 x 10^7^ | 3.6 x 10^7^ | BD | 7.1 x 10^7^ |
| EPP | BD | BD | BD | 8.3 x 10^8^  ab | BD | 1.3 x 10^8^  a | 1.9 x 10^9^  b | 7.2 x 10^8^  a |
| 10 dpi, qPCR | | | | | | | | |
| EPF | BD | 1.1 x 10^7^  a | BD | BD | 1.2 x 10^7^  ab | BD | 3.2 x 10^4^  bc | BD  c |
| EPN | BD | BD | 1.3 x 10^2^ | BD | 2.9 x 10^1^ | BD | BD | BD |
| NB | BD | BD | 7.2 x 10^7^  a | BD | 1.7 x 10^7^  a | 2.6 x 10^7^  a | BD | 2.2 x 10^7^  b |
| EPP | 3.9 x 10^5^ | BD | BD | 3.6 x 10^6^  a | 1.3 x 10^5^ | 2.1 x 10^8^  b | 7.3 x 10^8^  b | 2.3 x 10^8^  b |
| Data shown are the mean colonisation of LCW larvae by EPF *M. brunneum* Bip5-gfp, EPN *S. feltiae* RS5-mche, NB *Xenorhabdus* sp. SM5-mcherry and EPP *P. chlororaphis* PCLRT03-mturq as determined at 1, 5, and 10 dpi (n = 6 per time-point) by selective plating (in cfu per larva) and qPCR (in units per larva), respectively, in LCW repetition 5. Treatments: control = no BCA application, P = EPP *P. chlororaphis* PCLRT03-mturq, N = EPN *S. feltiae* RS5-mche, F = EPF *M. brunneum* Bip5-gfp, FN, PN, PF and PFN = double and triple combinations of respective BCA. BD = below detection. Letters in the second line refer to significant differences at *P* < 0.05 according to a TukeyHSD test. | | | | | | | | |

| **Table S6** Colonisation of *D. balteata* (BCB) larvae by EPF, EPN, NB and EPP | | | | | | | | |
| --- | --- | --- | --- | --- | --- | --- | --- | --- |
| Treat | control | F | N | P | FN | PN | PF | PFN |
| 1 dpi, selective plating | | | | | | | | |
| EPF | BD | 1.6 x 10^2^ | BD | BD | 3.0 | BD | 3.3 x 10^1^ | 5.7 x 10^1^ |
| NB | BD | BD | BD | BD | 2.0 x 10^2^ | BD | BD | BD |
| EPP | BD | BD | BD | 7.0 x 10^2^ | BD | 9.7 x 10^2^ | 6.7 x 10^1^ | 1.7 x 10^4^ |
| 3 dpi, selective plating (EPF, NB, EPP) or qPCR (EPN) | | | | | | | | |
| EPF | BD | 4.4 x 10^2^  a | BD | BD | 3.9 x 10^1^  ab | BD | 2.5 x 10^2^  b | 2.2 x 10^1^  ab |
| EPN | BD | NA | 2.6 x 10^3^ | 1.6 x 10^3^ | 1.8 x 10^3^ | 4.6 x 10^3^ | BD | 2.7 x 10^2^ |
| NB | BD | BD | 8.1 x 10^3^ | 2.0 | 1.5 x 10^5^ | 1.1 x 10^5^ | BD | 2.3 x 10^4^ |
| EPP | BD | 2.5 x 10^2^ | 2.0 | 3.1 x 10^3^ | BD | 1.7 x 10^4^ | 1.9 x 10^6^ | 2.0 x 10^4^ |
| 5 dpi, qPCR | | | | | | | | |
| EPF | BD | 1.4 x 10^7^ | BD | BD | 8.8 x 10^5^ | BD | 2.6 x 10^7^ | 3.4 x 10^5^ |
| EPN | BD | BD | 3.1 x 10^2^ | BD | 1.2 x 10^2^ | 6.0 x 10^2^ | BD | 3.7 x 10^1^ |
| NB | BD | BD | 5.8 x 10^7^ | BD | 2.0 x 10^7^ | 4.6 x 10^7^ | BD | 1.0 x 10^7^ |
| EPP | BD | 7.1 x 10^4^ | BD | 2.4 x 10^8^ | 2.4 x 10^4^ | 3.8 x 10^8^ | 2.2 x 10^8^ | 9.5 x 10^7^ |
| 7 dpi, qPCR | | | | | | | | |
| EPF | BD | 5.8 x 10^7^ | BD | BD | 9.8 x 10^4^ | BD | 8.7 x 10^4^ | 4.4 x 10^4^ |
| EPN | BD | BD | 1.3 x 10^5^ | 1.7 x 10^2^ | 4.6 x 10^4^ | 8.9 x 10^2^ | BD | 5.7 x 10^2^ |
| NB | BD | BD | 7.7 x 10^6^  a | BD | 2.0 x 10^5^  ab | 7.5 x 10^5^  b | BD | 2.0 x 10^5^  ab |
| EPP | BD | BD | BD | 1.2 x 10^8^ | BD | 4.6 x 10^7^ | 2.4 x 10^8^ | 1.5 x 10^8^ |
| 10 dpi, qPCR | | | | | | | | |
| EPF | BD | BD | BD | BD | BD | BD | 5.9 x 10^4^ | 1.1 x 10^5^ |
| EPN | BD | BD | 7.0 x 10^2^ | BD | 9.0 | 2.8 x 10^2^ | 2.0 | 9.0 |
| NB | BD | BD | 2.8 x 10^7^ | BD | 1.4 x 10^6^ | 3.7 x 10^7^ | 1.5 x 10^4^ | 3.2 x 10^7^ |
| EPP | BD | 1.7 x 10^5^ | BD | 2.0 x 10^7^ | BD | 9.0 x 10^7^ | 2.7 x 10^7^ | 2.4 x 10^8^ |
| Data shown are mean colonisation of BCB larvae by EPF *M. brunneum* Bip5-gfp, EPN *S. feltiae* RS5-mche, NB *Xenorhabdus* sp. SM5-mcherry and EPP *P. chlororaphis* PCLRT03-mturq as determined at 1 dpi (n = 3), 3 dpi (n = 8), 5, 7 and 10 dpi (n=6) by selective plating (in cfu per larva) and qPCR (in units per larva), respectively, in BCB repetition 2. Treatments: control = no BCA application, P = EPP *P. chlororaphis* PCLRT03-mturq, N = EPN *S. feltiae* RS5-mche, F = EPF *M. brunneum* Bip5-gfp, FN, PN, PF and PFN = double and triple combinations of respective BCA. BD = below detection. Letters in the second line refer to significant differences at *P* < 0.05 according to a TukeyHSD test. | | | | | | | | |

# Supplementary Figures

|  |
| --- |
| **Fig. S1** Survival of *P. brassicae* (LCW) larvae after time-shift applications of EPP and EPN in repetition 3.  Treatments: control = no BCA application, N = EPN *S. feltiae* RS5, N t-6h = EPN applied 6 h earlier, P = EPP *P. chlororaphis* PCLRT03-gfp, PN t-6h = EPN applied 6 h before EPP, PN t0 = EPN and EPP applied simultaneously, PN t+6h = EPN applied 6 h after EPP. Different letters indicate significant differences at *P* < 0.05 according to a pairwise survival difference. Mean survival time and final mortality of this experiment are displayed in Table S2. |

|  |
| --- |
| **Fig. S2** Survival of *P. brassicae* (LCW) larvae after infection with single and combined applications of EPP, EPF and EPN in LCW repetition 3.  Treatments: control = no BCA application, P = EPP *P. chlororaphis* PCLRT03-gfp, N = EPN *S. feltiae* RS5, F = EPF *M. brunneum* Bip5, FN, PN, PF and PFN = double and triple combinations of respective BCA. Different letters indicate significant differences at *P* < 0.05 according to a pairwise survival difference. Mean survival time and final mortality of this experiment are displayed in Table S3. |

|  |
| --- |
| **Fig. S3** Survival of *P. brassicae* (LCW) larvae after infection with single and combined applications of EPP, EPF and EPN in LCW repetition 5.  Treatments: control = no BCA application, P = EPP *P. chlororaphis* PCLRT03-mturq, N = EPN *S. feltiae* RS5-mche, F = EPF *M. brunneum* Bip5-gfp, FN, PN, PF and PFN = double and triple combinations of respective BCA. Different letters indicate significant differences at *P* < 0.05 according to a pairwise survival difference. Mean survival time and final mortality of this experiment are displayed in Table S3. |

|  |
| --- |
| **Fig. S4** Survival of *D. balteata* (BCB) larvae after infection with single and combined applications of EPP, EPF and EPN in BCB repetition 2.  Treatments: control = no BCA application, P = EPP *P. chlororaphis* PCLRT03-mturq, N = EPN *S. feltiae* RS5-mche, F = EPF *M. brunneum* Bip5-gfp, FN, PN, PF and PFN = double and triple combinations of respective BCA. Different letters indicate significant differences at *P* < 0.05 according to a pairwise survival difference. Mean survival time and final mortality of this experiment are displayed in Table S4. |
